# Development and Validation of a Machine Learning‑Based Predictive Model for Assessing the Risk of Comorbid Depression in Patients With Asthma

**DOI:** 10.31083/AP47754

**Published:** 2025-10-28

**Authors:** Qiu Nie, Xu Deng, Xin Chen, Tianwei Lai, Wen Li, Yutong Liu, Jingyi Lin, Qingsong Ren, Jingjing Liu, Yinxu Wang, Yulei Xie

**Affiliations:** ^1^Department of Rehabilitation Medicine, Affiliated Hospital of North Sichuan Medical College, 637000 Nanchong, Sichuan, China; ^2^Department of Digestive Endoscopy Center, Digestive Disease Center, Suining Central Hospital, 629000 Suining, Sichuan, China; ^3^College of Sports Medicine and Rehabilitation, North Sichuan Medical College, 637000 Nanchong, Sichuan, China; ^4^Department of Rehabilitation Engineering, China Rehabilitation Science Institute, 100037 Beijing, China

**Keywords:** asthma, depression, machine learning, predictive model

## Abstract

**Objective:**

The aim of this study was to develop and validate a machine learning model to predict the risk of comorbid depression in asthma patients.

**Methods:**

We conducted a retrospective study of 2464 asthma patients with comorbid depression using National Health and Nutrition Examination Survey (NHANES) data. Feature selection was conducted using the Boruta algorithm and the Least Absolute Shrinkage and Selection Operator (LASSO). Eight machine learning algorithms, namely Decision Tree (DT), k-Nearest Neighbors (KNN), Light Gradient Booster Machine (LGBM), Logistic Regression (LR), Random Forest (RF), Support Vector Machine (SVM), eXtreme Gradient Boosting (XGBoost), and Multilayer Perceptron (MLP), were trained using 5-fold cross-validation methodology. Model performance was evaluated through various metrics such as area under the curve (AUC), accuracy, sensitivity, specificity, F1 score, and decision curve analysis (DCA). Interpretation was conducted using SHapley Additive exPlanations (SHAP) analysis, highlighting feature importance.

**Results:**

The training set comprised 1724 participants, while the validation set included 740 participants, with a depression prevalence of 14.45%. Significant predictors identified included hypertension, chronic obstructive pulmonary disease (COPD), stroke, sleep questionnaire (SLQ) scores, smoking status, Poverty Index Ratio (PIR), and educational level. The XGBoost model demonstrated superior performance compared with alternative machine learning (ML) algorithms, achieving an AUC of 0.750, an accuracy of 69.1%, a sensitivity of 68.2%, a specificity of 73.8%, and an F1 score of 79%. The SHAP method identified SLQ, PIR, and education level as the primary decision factors influencing the ML model’s predictions.

**Conclusion:**

The XGBoost model effectively predicts the risk of depression in asthma patients, serving as a valuable reference for early clinical identification and intervention.

## Main Points

∙ Hypertension, chronic obstructive pulmonary disease (COPD), stroke, sleep questionnaire (SLQ), smoking status, PIR, and educational attainment can significantly predict the risk of asthma accompanied by depression. The prevalence rate of depression among asthma patients is 14.45%.

∙ The eX-treme Gradient Boosting (XGBoost) prediction model showed the best performance in assessing the depression risk of asthma patients, with an area under the curve (AUC) of 0.750.

∙ The SHapley Additive exPlanations (SHAP) method showed that SLQ, Poverty Index Ratio (PIR), and educational level are the most crucial predictive factors for predicting depression in asthma patients.

## 1. Introduction

Bronchial asthma is a chronic inflammatory airway disease that impacts 262 million people globally [1]. In the United States, approximately 26 million asthmatics are afflicted by this disease, and is accompanied by more than 3000 deaths and a significant health burden of around $81 billion annually [2, 3, 4]. Asthma not only severely affects patients’ quality of life but also triggers psychiatric symptoms. Up to 71.4% of patients experience poorer quality of life and are more prone to psychiatric manifestations such as anxiety and depression [5, 6, 7]. Among patients with asthma and depression, 40% will exhibit symptoms such as poor sleep and insomnia [8, 9]. These issues not only affect the efficacy of asthma control but also lead to refractory asthma, increase the healthcare burden, significantly reduce the quality of life, and raise the risk of death [8]. Studies have indicated that asthma-associated depression is closely related to low-income families, smoking, chronic obstructive pulmonary disease (COPD), hypertension, and stroke [5, 10, 11]. However, there is currently limited research on these risk factors, and clinical predictive models have not been adequately developed. Therefore, it is recommended that a risk prediction model for depression in asthma patients be developed to enhance the management of these risk factors, to reduce the incidence of these co-morbidities.

Machine learning (ML), as a powerful predictive tool, has been widely used in fields such as medicine and engineering [12]. Its predictive accuracy surpasses that of traditional statistical methods [13]. While previous study have developed simple regression predictive models, there is a notable lack of predictive model platforms, which limits their clinical practicality [14]. ML-based algorithms can analyze extensive datasets and uncover subtle predictive risk factors, thereby enhancing clinical predictive capabilities.

This study utilized the dataset of the National Health and Nutrition Examination Survey (NHANES, 2005–2020) to establish machine learning models for predicting the risk of asthma accompanied by depression. A comprehensive machine learning model construction process, including preprocessing, feature selection, model training, and evaluation, was designed. Eight machine learning models were compared to select the model with the best performance as the final prediction model. The aim was to establish an effective prediction platform based on machine learning to provide a theoretical basis for clinical practice and improve the overall health management of the risk of asthma with depression.

## 2. Methods

### 2.1 Research Design

All participant information was collected independently by two researchers from the NHANES database (2005–2020), which is part of the Centers for Disease Control and Prevention (CDC) in the United States. The was a retrospective cohort study.

### 2.2 Data Acquisition

NHANES is a national programme to assess the health and nutritional status of adults and children living in the communities in the United States. The NHANES database collects and stores information on interviews and examinations and is updated every two years [15]. The Ethics Review Board of the National Center for Health Statistics approved this study. Written informed consent was obtained from participants prior to their inclusion in the NHANES database. Full details of the ethical application and written informed consent are available on the NHANES website (https://www.cdc.gov/nchs/nhanes). All continuous variables were standardized, while categorical variables were processed using categorical coding to ensure data consistency and usability [11]. Two researchers independently extracted information from the database on January 1, 2024. Pertinent data collected were as follows: Participant demographic data: age, sex (male and female), and race (Mexican American, Other Hispanic, Non-Hispanic White, Non-Hispanic Black and Other Race). Examination data: glucose (GLU), high-density lipoprotein cholesterol (HDL-C), low-density lipoprotein cholesterol (LDL-C), total cholesterol (TC), triglycerides (TG), triglyceride-glucose index (TyG). Questionnaire data: marital (married/living with partner, widowed/divorced/separated, never married), diabetes (yes or no), hypertension (yes or no), heart failure (yes or no), COPD (yes or no), coronary artery disease (CAD) (yes or no), cancer (yes or no), stroke (yes or no), sleep disorders (SLQ) (yes or no), body mass index (BMI) status (under weight, normal weight, over weight), smoking (never smoker, former smoker, now smoker), ratio of income to poverty (PIR) (non-low-income, low-income), education (less than 9th grade, 9–11th grade, high school graduate, some college or AA degree, college graduate or above), drinking (1 to 5 drinks/month, 5 to 10 drinks/month, ≥10 drinks/month).

### 2.3 Inclusion Criteria

**Fig. 1.**
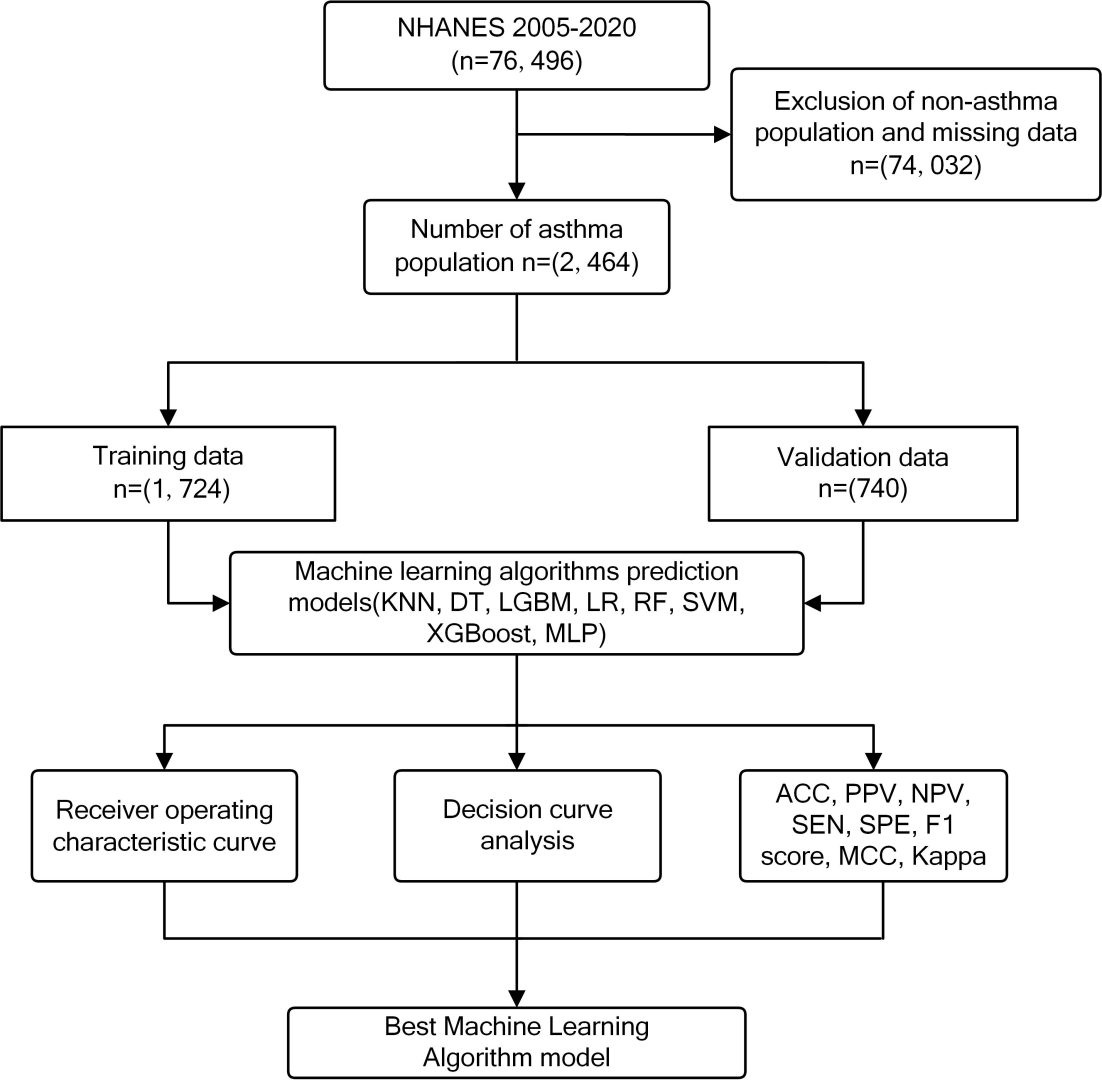
**Flow chart of the study**. NHANES, National Health and Nutrition Examination Survey; KNN, k-Nearest Neighbor Algorithm; DT, Decision Tree; LGBM, Light Gradient Booster Machine; LR, Logistic Regression; RF, Random Forest; SVM, Support Vector Machine; XGBoost, eXtreme Gradient Boosting; MLP, Multilayer Perceptron; ACC, accuracy; PPV, positive predictive value; NPV, negative predictive value; SEN, sensitivity; SPE, specificity; MCC, matthews correlation coefficient.

**Fig. 2.**
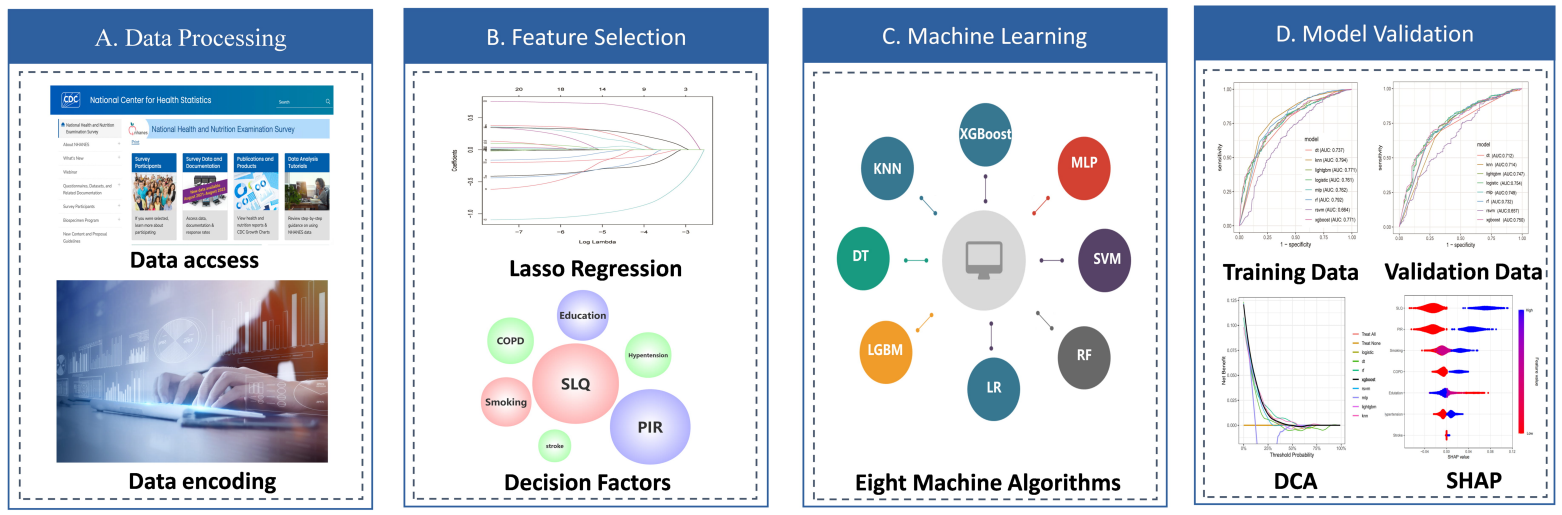
**Workflow for development and validation of the machine learning (ML) for predictions of the risk depression in patients with asthma**. (A) Data accsess and encoding. (B) Using LASSO regression for feature selection of predictors. (C) Eight machine learning algorithms were used for model training and development. (D) 5-fold cross-validation was used to evaluate the model. DCA, decision curve analysis; SHAP, SHapley Additive exPlanations; SLQ, sleep questionnaire; PIR, ratio of income to poverty; COPD, chronic obstructive pulmonary disease.

During the process of data merging, cleaning and organizing, individual data were longitudinally linked through the unique identified sequence number (SEQN). Variables with missing values exceeding 20% were excluded. Meanwhile, the data with missing values were also deleted. We only analyzed the complete data. Participants needed to be 20 years old or older. They had to partake in the dietary interview, undergo fasting glucose and triglyceride tests. They were required to complete the self-reported asthma information. Owing to the exclusion criteria, which accounted for the non-asthma population and missing data, the sample size was reduced 74,032. Eventually, a total of 2464 individuals with asthma were included and randomly divided into training and validation groups in a 7:3 ratio [16] (Fig. 1). Subsequently, a comparative analysis of variables was carried out. Among them, 1724 participants were assigned to the training data, and 740 participants were assigned to the validation data. The workflow of this study is shown in Fig. 2.

### 2.4 Assessment of Depression

The frequency of depressive symptoms in the past two weeks was assessed in participants by the Patient Health Questionnaire (PHQ-9) [17]. This screening tool is based on the criteria of the Diagnostic and Statistical Manual of Depression, Fourth Edition (DSM-IV) [18]. The PHQ-9 consists of 10 questions. After answering all 9 questions in full, a total score is calculated, ranging from 0 to 27. Higher scores indicate more severe depressive symptoms. Individuals with a total score ≥10 are considered to have moderate to severe depression. This threshold has a sensitivity of 88% and a specificity of 88% [19]. In the present study, for each participant with a total score ≥10, was characterized as having depression [17].

### 2.5 Definition of Asthma

Asthma was defined based on information from the NHANES questionnaire. A participant was considered to have asthma if they answered ‘yes’ to the question, ‘Has a doctor or other health care professional ever told you that you have asthma?’ The definition of asthma was defined if the subject answered ‘yes’ to the question, ‘Has a doctor or other health care professional ever told you that you have asthma?’ and also answered ‘yes’ to any of the following questions [20, 21, 22]:

(1) Have you had an asthma attack or flare-up in the last 12 months?

(2) In the past 12 months, have you visited an emergency room or urgent care center for asthma?

(3) In the past 3 months, have you taken any medication prescribed by a doctor for asthma?

(4) In the past 12 months, have you experienced wheezing or chest pain?

(5) In the past 12 months, have you taken medication prescribed by a doctor for wheezing or chest pain?

## 3. Statistical Analysis

Data analysis was conducted using R version 4.3.3 (R Foundation for Statistical Computing, Vienna, Austria; http://www.R-project.org). All data were assessed for normality. Continuous variables that followed a normal distribution were reported as mean ± standard deviation and analyzed using *t*-tests, while those that did not conform to normal distribution were presented as the median interquartile range (IQR). Categorical variables were expressed as counts and percentages of patients, and analyzed using the χ^2^ test, with a significance level set at *p* < 0.05 (two-sided).

In the study, eight different machine learning models were used for training and testing: Decision Tree (DT), k-Nearest Neighbor Algorithm (KNN), Light Gradient Booster Algorithm (LGBM), Logistic Regression (LR), Random Forest (RF), Support Vector Machines (SVM), eXtreme Gradient Boosting (XGBoost), and Multilayer Perceptron (MLP). The dataset was divided in a 7:3 ratio, with 70% allocated to the training set and 30% to the validation set. Internal five-fold cross-validation was used, and the evaluation metrics included area under the curve (AUC) and decision curve analysis (DCA). AUC measures the model’s discriminative ability, while DCA assesses the net benefit threshold of the predictions. Additionally, the SHapley Additive exPlanations (SHAP) method was utilized to determine the importance of each variable, highlighting their relative contributions to the model. To facilitate the integration of machine learning into clinical practice, the research team created a predictive modeling website for assessing the risk of asthma-associated depression, accessible at (https://chunyuzhang28.shinyapps.io/asthma/).

## 4. Results

### 4.1 Baseline Data

A total of 2464 individuals with asthma were included in the analysis and randomly divided into a training set of 1724 (70%) and a validation set of 740 (30%) (Fig. 1). The participants had an average age of 47.66 ± 17.51 years, with 42.57% being male and 57.43% female. Among them, 14.45% were diagnosed with depression, while 85.55% did not have this condition. The average GLU level was 110.27 ± 37.37, the average HDL-C was 1.39 ± 0.43, the average LDL-C was 111.37 ± 36.01, the average TC was 188.52 ± 41.38, the average TG level was 116.16 ± 66.12, and the average TyG was 8.58 ± 0.65. In terms of relationship status, 54.59% were married or living with a partner. Regarding comorbidities, 20.86% had diabetes, 44.85% had hypertension, 6.21% had heart failure, 24.76% had COPD, 88.92% had cancer, 5.40% had stroke, and 26.38% had SLQ. The prevalence of obesity based on was 48.09%, and the current smoking rate was 24.63%. Additionally, 34.94% of the participants came from low-income families, and 45.82% were non-Hispanic whites, which was the highest proportion among ethnic groups. In terms of educational attainment, 35.80% had some college education or an associate’s degree, representing the highest subgroup. Regarding alcohol consumption, 54.06% drank 1 to <5 drinks per month, which was the most prevalent drinking pattern. Notably, the distributions of these variables did not show significant differences between the training and internal test groups, with the exceptions of LDL-C and TC (Table 1). The proportion of depressed patients was significantly higher than that of non-depressed patients among those with conditions such as GLU, TG, TyG, alongside demographic factors like marital status (married/living with a partner), diabetes, hypertension, heart failure, COPD, CAD, cancer, stroke, obesity, smoking, low income, non-Hispanic white ethnicity, and having attended some college or obtained an AA degree. Detailed results are provided in Table 2.

**Table 1.** **Patient demographics and baseline characteristics in training cohort and test cohort**.

Variables		Total (n = 2464)	Training Cohort	Test Cohort	Statistic	*p*
			(n = 1724)	(n = 740)		
Age (years), Mean ± SD		47.66 ± 17.51	47.58 ± 17.43	47.85 ± 17.71	t = 0.36	0.718
GLU (mg/dL), Mean ± SD		110.27 ± 37.37	110.20 ± 36.91	110.42 ± 38.43	t = 0.14	0.892
HDL-C (mmol/L), Mean ± SD		1.39 ± 0.43	1.39 ± 0.43	1.40 ± 0.42	t = 0.58	0.560
LDL-C (mg/dL), Mean ± SD		111.37 ± 36.01	112.37 ± 36.72	109.03 ± 34.22	t = –2.11	0.035
TC (mg/dL), Mean ± SD		188.52 ± 41.38	189.70 ± 42.61	185.80 ± 38.24	t = –2.24	0.025
TG (mg/dL), Mean ± SD		116.16 ± 66.12	117.69 ± 67.89	112.59 ± 61.70	t = –1.76	0.079
TyG, Mean ± SD		8.58 ± 0.65	8.59 ± 0.65	8.56 ± 0.64	t = –1.16	0.245
Depression, n (%)					χ² = 0.40	0.525
	No	2108 (85.55)	1480 (85.85)	628 (84.86)		
	Yes	356 (14.45)	244 (14.15)	112 (15.14)		
Gender, n (%)					χ² = 0.13	0.719
	Male	1049 (42.57)	738 (42.81)	311 (42.03)		
	Female	1415 (57.43)	986 (57.19)	429 (57.97)		
Martial, n (%)					χ² = 1.64	0.441
	Married/Living with Partner	1345 (54.59)	927 (53.77)	418 (56.49)		
	Widowed/Divorced/Separated	579 (23.50)	410 (23.78)	169 (22.84)		
	Never married	540 (21.92)	387 (22.45)	153 (20.68)		
Diabetes, n (%)					χ² = 2.51	0.113
	No	1950 (79.14)	1379 (79.99)	571 (77.16)		
	Yes	514 (20.86)	345 (20.01)	169 (22.84)		
Hypertension, n (%)					χ² = 1.15	0.283
	No	1359 (55.15)	963 (55.86)	396 (53.51)		
	Yes	1105 (44.85)	761 (44.14)	344 (46.49)		
Heart Failure, n (%)					χ² = 0.14	0.709
	No	2311 (93.79)	1619 (93.91)	692 (93.51)		
	Yes	153 (6.21)	105 (6.09)	48 (6.49)		
COPD, n (%)					χ² = 0.03	0.854
	NO	1854 (75.24)	1299 (75.35)	555 (75.00)		
	Yes	610 (24.76)	425 (24.65)	185 (25.00)		
CAD, n (%)					χ² = 0.19	0.665
	No	2351 (95.41)	1647 (95.53)	704 (95.14)		
	Yes	113 (4.59)	77 (4.47)	36 (4.86)		
Cancer, n (%)					χ² = 0.964	0.326
	Yes	273 (11.08)	184 (10.67)	89 (12.03)		
	No	2191 (88.92)	1540 (89.33)	651 (87.97)		
Stroke, n (%)					χ² = 0.04	0.837
	No	2331 (94.60)	1632 (94.66)	699 (94.46)		
	Yes	133 (5.40)	92 (5.34)	41 (5.54)		
SLQ, n (%)					χ² = 1.16	0.282
	No	1814 (73.62)	1280 (74.25)	534 (72.16)		
	Yes	650 (26.38)	444 (25.75)	206 (27.84)		
BMI status, n (%)					χ² = 0.64	0.888
	Under weight	43 (1.75)	29 (1.68)	14 (1.89)		
	Normal weight	529 (21.47)	372 (21.58)	157 (21.22)		
	Over weight	707 (28.69)	501 (29.06)	206 (27.84)		
	Obese	1185 (48.09)	822 (47.68)	363 (49.05)		
Smoking, n (%)					χ² = 0.86	0.649
	Never smoker	1186 (48.13)	838 (48.61)	348 (47.03)		
	Former smoker	671 (27.23)	470 (27.26)	201 (27.16)		
	Now smoker	607 (24.63)	416 (24.13)	191 (25.81)		
PIR, n (%)					χ² = 0.35	0.554
	Non-low-income	1603 (65.06)	1128 (65.43)	475 (64.19)		
	Low-income	861 (34.94)	596 (34.57)	265 (35.81)		
Race, n (%)					χ² = 3.97	0.410
	Mexican American	209 (8.48)	149 (8.64)	60 (8.11)		
	Other Hispanic	243 (9.86)	164 (9.51)	79 (10.68)		
	Non-Hispanic White	1129 (45.82)	789 (45.77)	340 (45.95)		
	Non-Hispanic Black	634 (25.73)	436 (25.29)	198 (26.76)		
	Other Race	249 (10.11)	186 (10.79)	63 (8.51)		
Education, n (%)					χ² = 4.43	0.351
	Less than 9th grade	149 (6.05)	96 (5.57)	53 (7.16)		
	9–11th grade	341 (13.84)	229 (13.28)	112 (15.14)		
	High school graduate	565 (22.93)	404 (23.43)	161 (21.76)		
	Some college or AA degree	882 (35.80)	621 (36.02)	261 (35.27)		
	College graduate or above	527 (21.39)	374 (21.69)	153 (20.68)		
ALQ, n (%)					χ² = 2.05	0.561
	Non-drinker	628 (25.49)	438 (25.41)	190 (25.68)		
	1 to <5 drinks/month	1332 (54.06)	939 (54.47)	393 (53.11)		
	5 to <10 drinks/month	173 (7.02)	113 (6.55)	60 (8.11)		
	≥10 drinks/month	331 (13.43)	234 (13.57)	97 (13.11)		

Note: t: *t*-test, χ^2^: Chi-square test, SD, standard deviation. Abbreviations: GLU, glucose; HDL-C, high-density lipoprotein cholesterol; LDL-C, ligh-density lipoprotein cholesterol; TC, total cholesterol; TG, triglycerides; TyG, triglyceride-glucose index; CAD, coronary artery disease; BMI, body mass index; ALQ, alcohol.

**Table 2.** **Demographics and clinical characteristics of study in the non-depression and depression patients**.

Variables		Non-depression	Depression	Statistic	*p*
		(n = 2108)	(n = 356)		
Age, Mean ± SD		47.45 ± 17.80	48.89 ± 15.63	t = –1.57	0.117
GLU (mg/dL), Mean ± SD		109.21 ± 35.71	116.50 ± 45.52	t = –2.88	0.004
HDL-C (mmol/L), Mean ± SD		1.40 ± 0.43	1.36 ± 0.41	t = 1.64	0.102
LDL-C (mg/dL), Mean ± SD		111.36 ± 35.60	111.42 ± 38.42	t = –0.03	0.976
TC (mg/dL), Mean ± SD		188.33 ± 41.13	189.67 ± 42.83	t = –0.56	0.574
TG (mg/dL), Mean ± SD		114.11 ± 64.75	128.32 ± 72.61	t = –3.47	<0.001
TyG, Mean ± SD		8.56 ± 0.64	8.72 ± 0.68	t = –4.37	<0.001
Gender, n (%)				χ² = 15.13	<0.001
	Male	931 (44.17)	118 (33.15)		
	Female	1177 (55.83)	238 (66.85)		
Martial, n (%)				χ² = 24.14	<0.001
	Married/Living with Partner	1176 (55.79)	169 (47.47)		
	Widowed/Divorced/Separated	459 (21.77)	120 (33.71)		
	Never married	473 (22.44)	67 (18.82)		
Diabetes, n (%)				χ² = 20.03	<0.001
	No	1700 (80.65)	250 (70.22)		
	Yes	408 (19.35)	106 (29.78)		
Hypertension, n (%)				χ² = 24.94	<0.001
	No	1206 (57.21)	153 (42.98)		
	Yes	902 (42.79)	203 (57.02)		
Heart Failure, n (%)				χ² = 5.52	0.019
	No	1987 (94.26)	324 (91.01)		
	Yes	121 (5.74)	32 (8.99)		
COPD, n (%)				χ² = 53.06	<0.001
	No	1641 (77.85)	213 (59.83)		
	Yes	467 (22.15)	143 (40.17)		
CAD, n (%)				χ² = 10.23	0.001
	No	2023 (95.97)	328 (92.13)		
	Yes	85 (4.03)	28 (7.87)		
Cancer, n (%)				χ² = 0.06	0.812
	No	1874 (88.90)	318 (89.33)		
	Yes	234 (11.10)	38 (10.67)		
Stroke, n (%)				χ² = 27.78	<0.001
	No	2015 (95.59)	316 (88.76)		
	Yes	93 (4.41)	40 (11.24)		
SLQ, n (%)				χ² = 119.54	<0.001
	No	1636 (77.61)	178 (50.00)		
	Yes	472 (22.39)	178 (50.00)		
BMI status, n (%)				χ² = 14.56	0.002
	Under weight	35 (1.66)	8 (2.25)		
	Normal weight	471 (22.34)	58 (16.29)		
	Over weight	619 (29.36)	88 (24.72)		
	Obese	983 (46.63)	202 (56.74)		
Smoking, n (%)				χ² = 68.64	<0.001
	Never smoker	1071 (50.81)	115 (32.30)		
	Former smoker	577 (27.37)	94 (26.40)		
	Now smoker	460 (21.82)	147 (41.29)		
PIR, n (%)				χ² = 100.95	<0.001
	Non-low-income	1455 (69.02)	148 (41.57)		
	Low-income	653 (30.98)	208 (58.43)		
Race, n (%)				χ² = 10.25	0.036
	Mexican American	188 (8.92)	21 (5.90)		
	Other Hispanic	194 (9.20)	49 (13.76)		
	Non-Hispanic White	965 (45.78)	164 (46.07)		
	Non-Hispanic Black	544 (25.81)	90 (25.28)		
	Other Race	217 (10.29)	32 (8.99)		
Education, n (%)				χ² = 49.38	<0.001
	Less than 9th grade	110 (5.22)	39 (10.96)		
	9-11th grade	271 (12.86)	70 (19.66)		
	High school graduate	476 (22.58)	89 (25.00)		
	Some college or AA degree	763 (36.20)	119 (33.43)		
	College graduate or above	488 (23.15)	39 (10.96)		
Drinking, n (%)				χ² = 4.42	0.219
	Non-drinker	523 (24.81)	105 (29.49)		
	1 to <5 drinks/month	1144 (54.27)	188 (52.81)		
	5 to <10 drinks/month	150 (7.12)	23 (6.46)		
	≥10 drinks/month	291 (13.80)	40 (11.24)		

### 4.2 Selection of Predictors

**Fig. 3.**
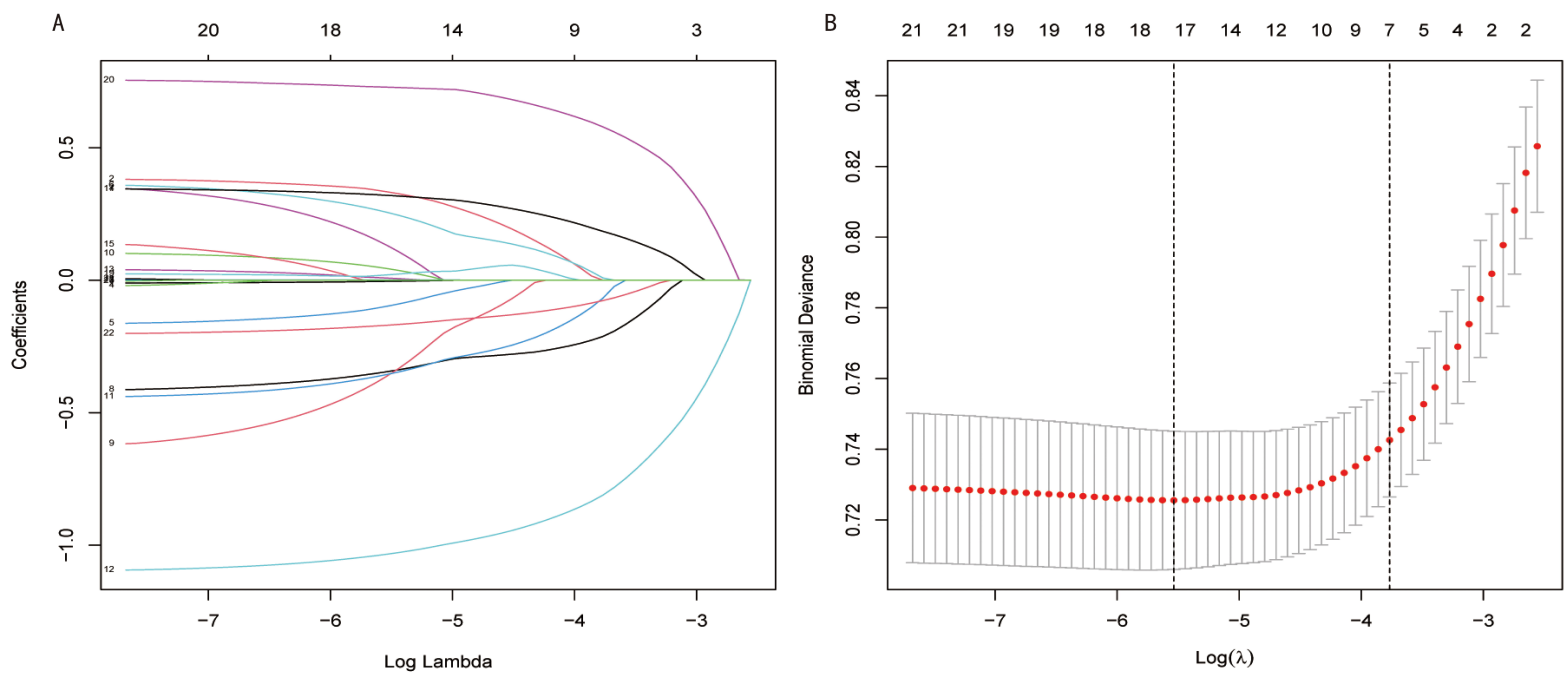
**Least Absolute Shrinkage and Selection Operator (LASSO) regression plot**. (A) Plot of LASSO coefficient profiles. (B) Plot of partial likelihood deviance.

Both groups exhibited statistically significant differences in terms of hypertension, COPD, stroke, SLQ, smoking, PIR, and education level (*p* < 0.05). The multifactorial logistic regression analysis revealed an odds ratio (OR) of 1.36 (1.05~1.75) for asthma combined with depression in comparison to non-hypertensive patients. Against the reference values, the ORs for asthma patients with concurrent depression were 1.48 (1.14~1.93) for COPD, 1.55 (1.01~2.37) for stroke, 3.08 (2.40~3.95) for SLQ, 1.98 (1.47~2.67) for smoking, and 2.20 (1.70~2.85) for PIR. Notably, higher education levels were linked to a reduced risk of depression, with an OR of 0.41 (95% CI: 0.24~0.70) (Table 3). The optimal subset of clinical characteristics identified through Lasso regression included SLQ, PIR, smoking, COPD, education, hypertension, and stroke, all of which were associated with the risk of depression in asthma patients (Fig. 3).

**Table 3.** **Univariate and multivariate logistic regression analysis**.

Variables		Univariate OR (95% CI)	*p*	Multivariate OR (95% CI)	*p*
Hypertension					
	No	1.00 (Reference)		1.00 (Reference)	
	Yes	1.77 (1.41∼2.23)	<0.001	1.36 (1.05∼1.75)	0.018
COPD					
	No	1.00 (Reference)		1.00 (Reference)	
	Yes	2.36 (1.86∼2.99)	<0.001	1.48 (1.14∼1.93)	0.003
Stroke					
	No	1.00 (Reference)		1.00 (Reference)	
	Yes	2.74 (1.86∼4.05)	<0.001	1.55 (1.01∼2.37)	0.046
SLQ					
	No	1.00 (Reference)		1.00 (Reference)	
	Yes	3.47 (2.75∼4.37)	<0.001	3.08 (2.40∼3.95)	<0.001
Smoking					
	Never smoker	1.00 (Reference)		1.00 (Reference)	
	Former smoker	1.52 (1.13∼2.03)	0.005	1.16 (0.85∼1.58)	0.351
	Now smoker	2.98 (2.28∼3.89)	<0.001	1.98 (1.47∼2.67)	<0.001
PIR					
	Non-low-income	1.00 (Reference)		1.00 (Reference)	
	Low-income	3.13 (2.49∼3.94)	<0.001	2.20 (1.70∼2.85)	<0.001
Education					
	Less than 9th grade	1.00 (Reference)		1.00 (Reference)	
	9–11th grade	0.73 (0.46∼1.14)	0.168	0.71 (0.44∼1.16)	0.169
	High school graduate	0.53 (0.34∼0.81)	0.004	0.60 (0.38∼0.95)	0.030
	Some college or	0.44 (0.29∼0.66)	<0.001	0.54 (0.34∼0.84)	0.007
	AA degree				
	College graduate	0.23 (0.14∼0.37)	<0.001	0.41 (0.24∼0.70)	0.001
	or above				

Abbreviations: OR, odds ratio.

### 4.3 Model Performance

**Fig. 4.**
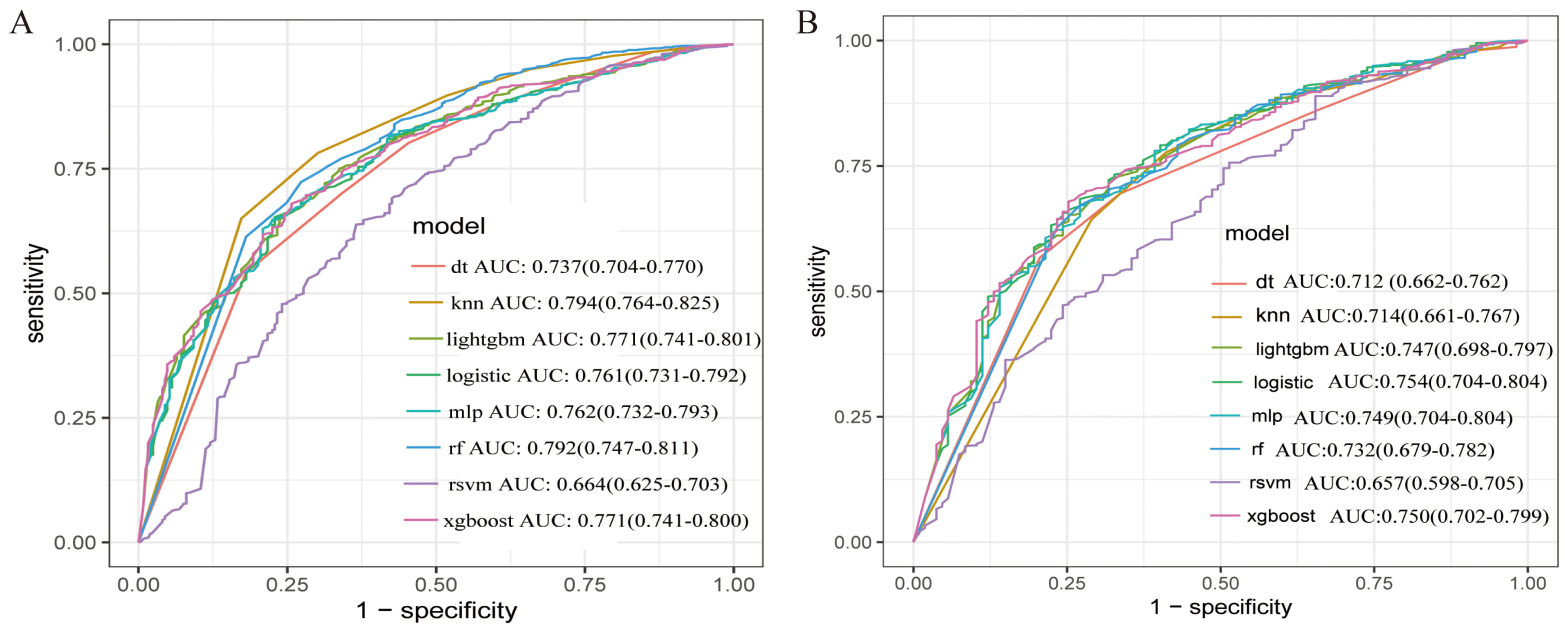
**Receiver operating characteristic curve (ROC) curve analysis of the eight machine learning algorithms for predicting short-term prognosis of asthma with depression patients**. (A) Training data. (B) Validation data.

The AUC values for the eight models in the training set are as follows: DT (0.737), KNN (0.794), light GBM (0.771), LR (0.761), MLP (0.762), RF (0.792), SVM (0.664), and XGBoost (0.771) (Fig. 4A). In the validation set, the AUC values are DT (0.712), KNN (0.714), light GBM (0.747), LR (0.754), MLP (0.749), RF (0.732), SVM (0.657), and XGBoost (0.750) (Fig. 4B). Overall, The XGBoost model demonstrated superior performance compared with alternative ML algorithms, achieving an AUC of 0.750, an accuracy of 69.1%, a sensitivity of 68.2%, a specificity of 73.8%, and an F1 score of 79% (Fig. 4 and Table 4).

**Table 4.** **Performance comparison of eight classification ML models**.

Characteristics	DT	KNN	LGBM	LR	RF	SVM	XGBoost	MLP
AUC	0.712	0.714	0.747	0.754	0.732	0.657	0.750	0.749
Accuracy	0.600	0.750	0.728	0.659	0.701	0.618	0.691	0.699
Sensitivity/recall	0.567	0.777	0.744	0.641	0.708	0.626	0.682	0.705
Specificity	0.794	0.589	0.636	0.766	0.664	0.579	0.738	0.664
PPV	0.942	0.918	0.924	0.942	0.926	0.898	0.939	0.925
NPV	0.237	0.309	0.296	0.265	0.277	0.207	0.282	0.275
F1 score	0.708	0.842	0.824	0.763	0.802	0.737	0.790	0.800
MCC	0.254	0.288	0.288	0.291	0.275	0.147	0.305	0.272
KAPPA	0.182	0.266	0.257	0.229	0.235	0.117	0.252	0.232

Abbreviations: AUC, area under the curve.

### 4.4 Decision Curve Analysis (DCA)

**Fig. 5.**
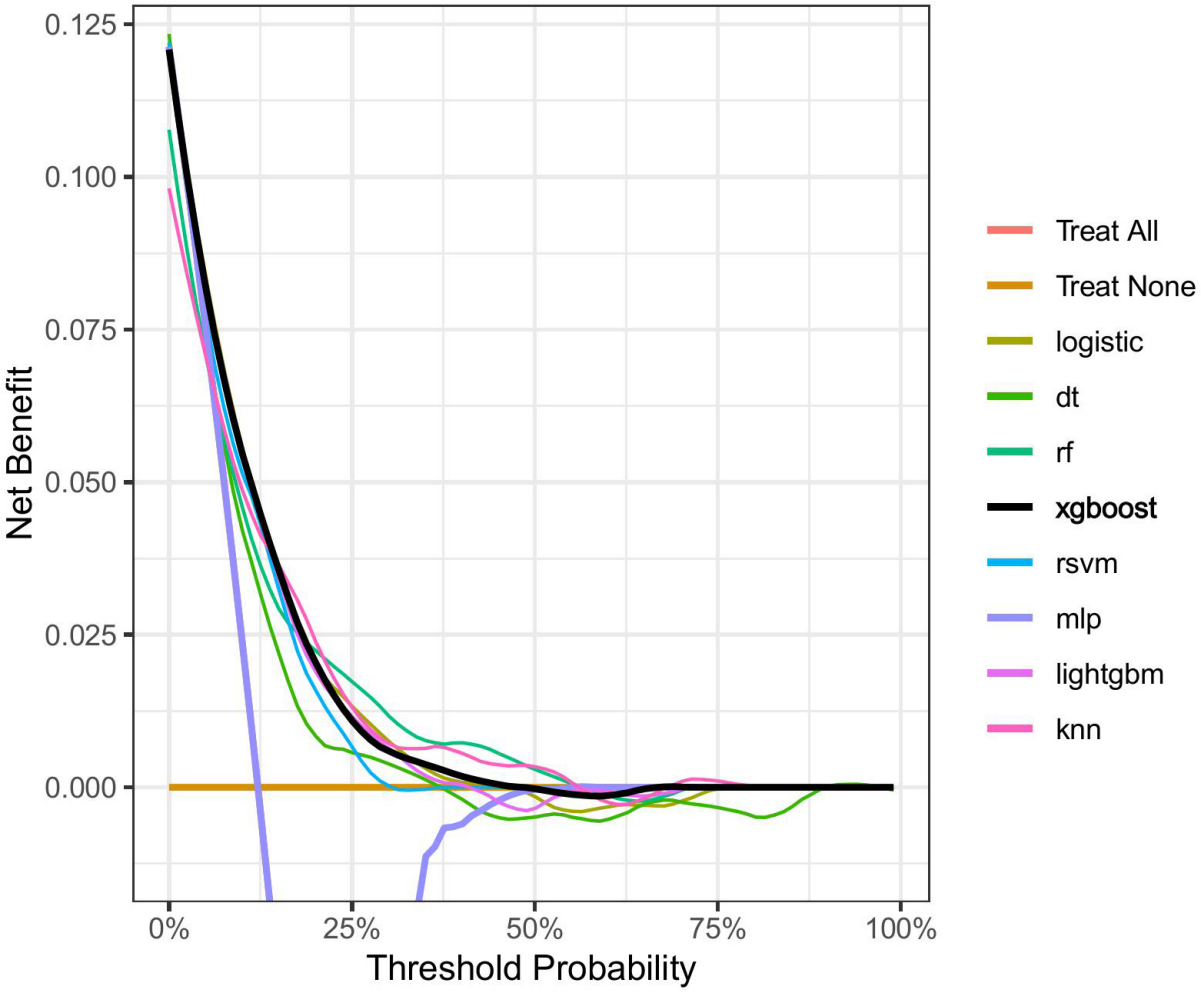
**Decision curves for each model**.

DCA was conducted on the eight machine learning models in the validation set to evaluate the net benefit of the best models in clinical decision-making, defined as the minimum probability of illness necessitating further intervention. Fig. 5 shows the net benefit at various threshold probabilities. The orange line represents the scenario where all patients receive the intervention, while the yellow line indicates all the patients do not receive it. Here, “Treat all” overlaps with “MLP”. Given the heterogeneous nature of the study population, the treatment strategy provided by any of the machine learning-based models outperformed the default strategy of intervention or no intervention for all patients, with the XGBoost model outperforming the other models in terms of net benefit.

### 4.5 SHAP-Based Model Interpretability Analysis

**Fig. 6.**
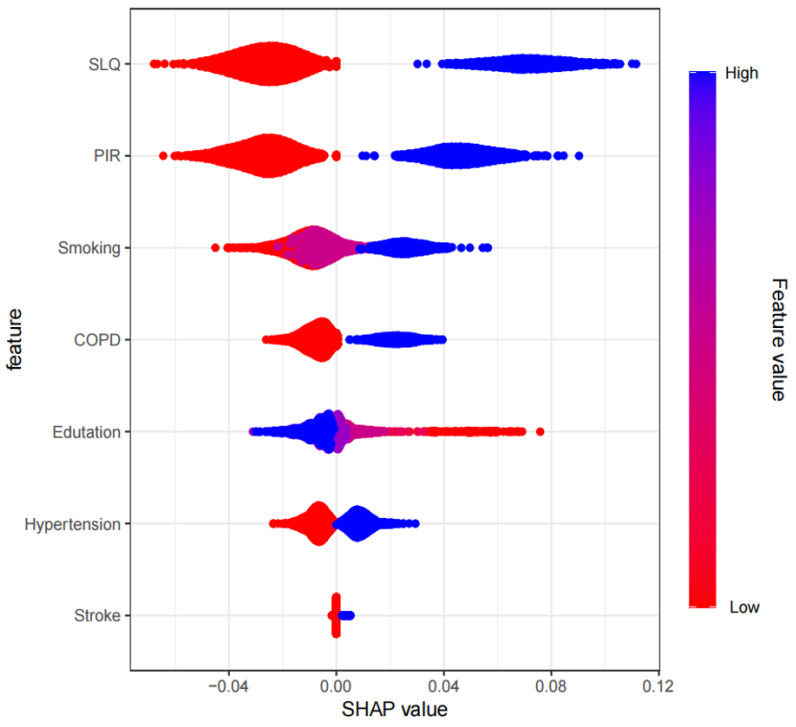
**SHAP dendrogram of features of the logistic regression model**.

**Fig. 7.**
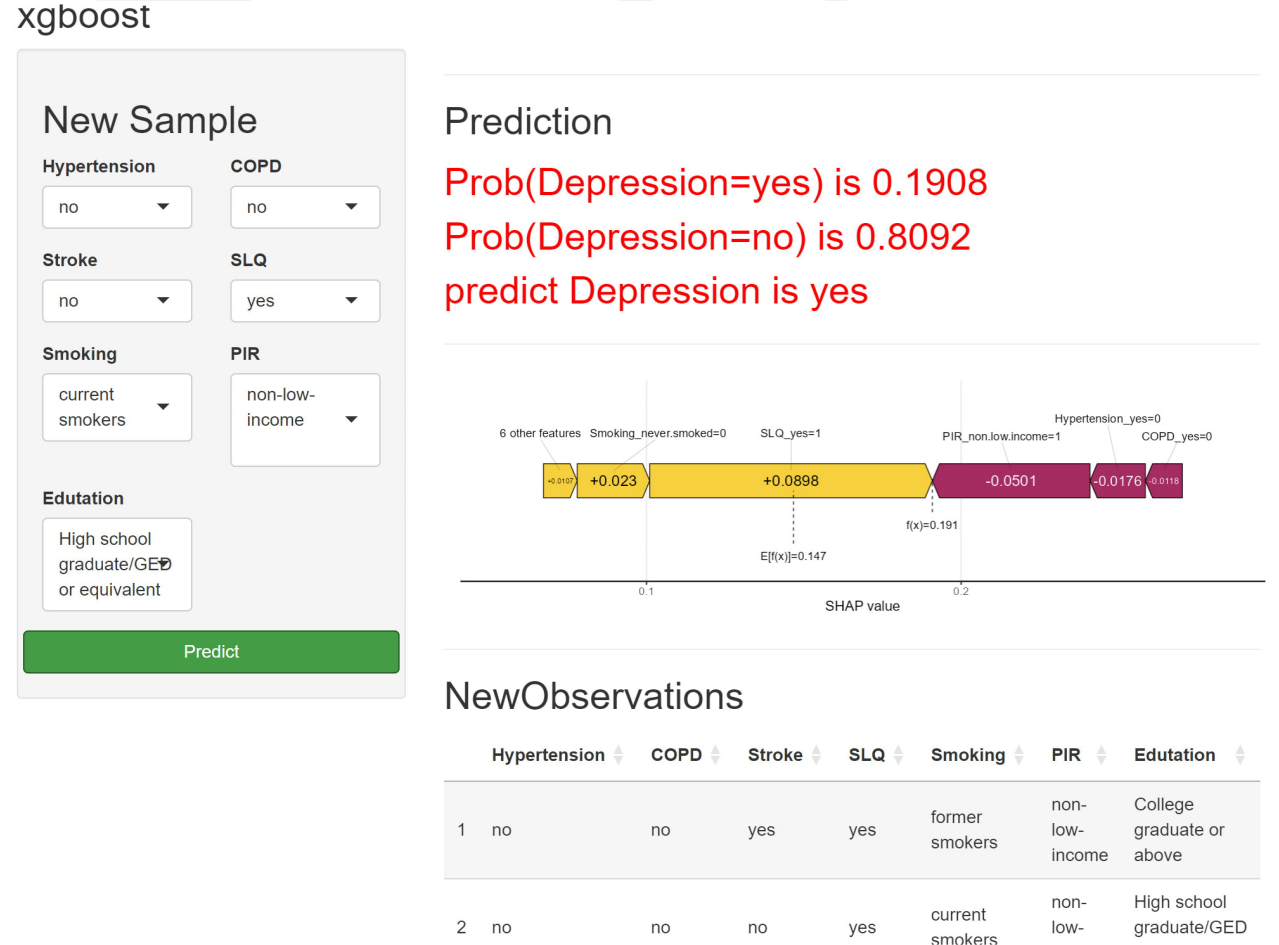
**XGBoost prediction model accessible at https://chunyuzhang28.shinyapps.io/asthma/**.

We visualized the importance of each predictor variable in the XGBoost model predictions using SHAP plots. Sleep disturbance emerged as the most significant predictor, followed by PIR and education level. The effect of each variable on the outcome is illustrated by the magnitude of the SHAP value (indicated by color changes) and the trend along the horizontal axis (which represents the probability of a poor outcome). For instance, individuals with severe sleep disorders (shown in blue) are more likely to experience depression than those without sleep disorders (shown in red). Similarly, those with lower household incomes face a higher risk of depression compared to individuals with higher incomes, and asthma patients with lower education levels may also have an increased risk of depression (Fig. 6). In addition, we have developed a predictive modeling website for evaluating the risk of asthma-associated depression. Medical staff can use this website to quickly assess the risk of depression in asthma patients within a short period of time. It can be accessed via the following link: (https://chunyuzhang28.shinyapps.io/asthma/) (Fig. 7). For instance, consider an asthma patient who does not suffer from hypertension, coronary heart disease, or stroke. This patient has a history of sleep disorders and smoking, comes from a high-income family, and belongs to a highly educated demographic group. Following the construction and application of a machine learning predictive model, it is determined that the probability of this individual having depression is 0.1908. Such a result implies a certain predisposition to depression, and thus clinicians can carry out targeted interventions on this patient based on this finding.

## 5. Discussion

Depression is a significant global mental health issue. Genetic, biological, and environmental factors can contribute to comorbid depression in asthma patients. Early prediction and intervention for depression in these individuals are particularly important. ML-based methods have demonstrated promising applications in the diagnosis and treatment of depression. In this study, we developed a predictive model for asthma-related comorbid depression and created an interactive visual prediction platform. Our goal was to offer personalized counseling and enhance medical decision-making, ultimately leading to more effective management strategies in clinical practice. Compared to traditional prediction models, such as nomogram plots, this platform provides more efficient support for clinical applications.

This study referred to previous studies on common risk factors associated with depression in asthma patients, including hypertension, COPD, stroke, SLQ, smoking, PIR and educational level [20, 23]. In a study of 100 patients with bronchial asthma, Zhang *et al*. [24] found that age ≥60 years, asthma control score <20, junior high school or lower education level, rural residence, asthma grade 4 and clinical stage were the main factors for comorbid anxiety and depression. These findings aligned with the results of the current study, based on multivariate retrospective analyses, which showed that hypertension, COPD, stroke, SLQ, smoking, PIR and lower education level increased the risk of comorbid depression in asthma, confirming previous findings.

Behavioral factors such as sleep disturbance and smoking are strongly associated with the onset of depression, particularly in people with asthma. Studies have shown that asthmatics with sleep disturbances are more likely to experience severe depressive symptoms [25, 26]. Approximately 90% of patients experience coughing or wheezing at night, which is associated with nocturnal bronchial hyperresponsiveness and decreased lung capacity [7, 25]. The prevalence of comorbid anxiety and depression is as high as 20% in patients with asthmatic insomnia [9, 27]. Rhoads *et al*. [5] showed that insomnia not only exacerbates anxiety symptoms in patients with asthma, but can also lead to depressive symptoms such as trouble falling asleep, difficulty staying asleep, and early awakening, which can further exacerbate depressive symptoms [28, 29]. In turn, chronic insomnia can lead to mood problems by affecting brain function and reducing mood regulation through mechanisms that may be related to an imbalance of neurotransmitters involved in mood regulation (e.g., serotonin, norepinephrine and dopamine) and may lead to abnormalities in cortisol secretion, which can induce chronic stress and depression [30]. Similarly, smoking, a risk factor for acute asthma exacerbations and limited irreversible airflow, not only exacerbates asthma symptoms but may also induce persistent limitation airflow [31]. Although the nicotine in smoke may improve mood in the short term, long-term smoking exacerbates asthma and depressive symptoms [32, 33]. Toyama *et al*. [34] analyzed 1962 asthma patients and found that smokers had a significantly higher prevalence of asthma than non-smokers and that depressed mood had a significant negative effect on asthma control scores. These findings highlight the importance of smoking cessation in the health management of asthma patients.

Socioeconomic factors such as PIR and education level play a key role in the health status of asthma patients [35]. Low-income families face greater economic pressures, including medical, educational and living expenses, and this chronic economic pressure increases the risk of depression in asthma patients [10]. Moreover, education level has a significant impact on managing depression in asthma patients. Tarraf *et al*. [36] found that less than 30% of asthma patients had good control and that the level of control was closely related to smoking frequency, lower educational level and inappropriate use of medication. Asthmatics with lower education levels often lack awareness of mental illness and effective coping strategies, making them more susceptible to negative emotions like anxiety and depression. Therefore, attention should be paid to the family income and the education level of asthma patients to reduce the risk of depression through early detection and prevention.

Disease factors such as COPD, hypertension and stroke may negatively affect the mental health of asthma patients and increase the risk of depression mood [11]. These chronic diseases not only lead to symptoms such as dyspnea, but also reduce the quality of life of patients, which increases the risk of depression. Chronic health problems may also increase psychological stress [37, 38]. Notably, these diseases may be interrelated, for example, patients with COPD are often associated with complications such as hypertension, which may further exacerbate depression. Therefore, it is recommended to enhance chronic disease management in patients with asthma to reduce the risk of depression.

XGBoost is an integrated decision tree-based learning algorithm widely used in risk prediction, feature selection and clinical decision support, which has the advantages of fast speed, good results, high flexibility and high predictive accuracy. In this study, it was found that the prediction model of XGBoost performed the best in terms of synthesis and performance compared to algorithms such as DT, KNN, LGBM, LR, RF, SVM, MLP, etc., and was especially optimal in predicting asthma-combined with depression. Li *et al*. [39] found that the Cat Boost model was suitable for depression screening in older adults using data from the China Health and Retirement Longitudinal Study (CHARLS). Su *et al*. [40] predicted risk factors for depression in older adults using eight ML models and found that self-rated health, marital status, arthritis, and number of cohabits were key factors. Hatton *et al*. [41] confirmed that the XGBoost model outperformed the traditional logistic regression model in predicting depression in elderly patients. Xia *et al*. [42] also confirmed that the prediction of depression by XGBoost was superior to that of DT, SVM, Multivariate Adaptive Regression Splines (MARS), Artificial Neural Network (ANN), BT, RF, KNN and other models, supporting the findings of this study.

In conclusion, prevention of depression in asthma patients should focus on those patients with sleep disturbances, lower family economic income and lower educational levels. XGBoost has significant advantages in predicting depression in asthma patients, and by incorporating seven independent risk factors, it helps in the prevention and management of depression improve their mental health.

## 6. Limitation

This study has several limitations: First, all participants were Americans aged ≥20 years and the findings may not be generalizable to other populations. Second, the diagnoses of asthma and depression in the NHANES data were based on participants’ self-reports, which might have retrospective biases and consequently affect the reliability of the results; Third, the lack of an external validation cohort in the NHANES data leads to the possibility that the applicability of the developed XGBoost model in clinical practice may be limited; Fourth, although the NHANES dataset covers a wide range of health and nutrition-related variables, it still does not include all potentially important variables, such as certain socioeconomic factors, environmental factors, or genetic background, which limits a comprehensive understanding of complex health issues. It is recommended that in the future, the XGBoost model be applied to asthma patients in different countries and regions to predict the risk of depression onset, so as to conduct internal and external validation of the model and improve its stability.

## 7. Conclusion

The interpretable XGBoost prediction model we developed performs optimally in assessing the risk of depression in asthma patients. This interpretable machine learning approach accurately identifies risk factors for depression in asthma patients and enhances physicians’ confidence in the prediction results, thus helping them to identify asthma patients at high risk of depression and provides them with the best treatment options.

## Availability of Data and Materials

These survey data are free and publicly available, and can be downloaded directly from the NHANES website (http://www.cdc.gov/nchs/nhanes.htm) by users and researchers worldwide.

## References

[1] (2022). The Global Asthma Report 2022. *The International Journal of Tuberculosis and Lung Disease: the Official Journal of the International Union against Tuberculosis and Lung Disease*.

[2] Granell R, Rodriguez S (2020). Confirmed causal effect of obesity on asthma and new insights on potential underlying shared genetic mechanisms. *The Journal of Allergy and Clinical Immunology*.

[3] Sharma V, Cowan DC (2021). Obesity, Inflammation, and Severe Asthma: an Update. *Current Allergy and Asthma Reports*.

[4] Nurmagambetov T, Kuwahara R, Garbe P (2018). The Economic Burden of Asthma in the United States, 2008-2013. *Annals of the American Thoracic Society*.

[5] Rhoads SL, Edinger J, Khatiwada A, Zimmer J, Zelarney P, Wechsler ME (2024). The impact of insomnia and depression on asthma control. *The Journal of Asthma: Official Journal of the Association for the Care of Asthma*.

[6] Palka JM, Peacock M, Tusken M, Ibrahim M, Najjab A, Carter L (2024). Depressive symptom severity in mid- to late life in individuals with and without asthma. *The Journal of Allergy and Clinical Immunology. in Practice*.

[7] Ali R, Ahmed N, Salman M, Daudpota S, Masroor M, Nasir M (2020). Assessment of Quality of Life in Bronchial Asthma Patients. *Cureus*.

[8] Vieira AA, Santoro IL, Dracoulakis S, Caetano LB, Fernandes ALG (2011). Anxiety and depression in asthma patients: impact on asthma control. *Jornal Brasileiro De Pneumologia: Publicacao Oficial Da Sociedade Brasileira De Pneumologia E Tisilogia*.

[9] Nutt D, Wilson S, Paterson L (2008). Sleep disorders as core symptoms of depression. *Dialogues in Clinical Neuroscience*.

[10] Huang K, Yang T, Xu J, Yang L, Zhao J, Zhang X (2019). Prevalence, risk factors, and management of asthma in China: a national cross-sectional study. *Lancet (London, England)*.

[11] Zhu M, Chen A (2024). Epidemiological characteristics of asthma-COPD overlap, its association with all-cause mortality, and the mediating role of depressive symptoms: evidence from NHANES 2005-2018. *BMC Public Health*.

[12] Stumpo V, Staartjes VE, Esposito G, Serra C, Regli L, Olivi A (2022). Machine Learning and Intracranial Aneurysms: From Detection to Outcome Prediction. *Acta Neurochirurgica. Supplement*.

[13] Sajjadian M, Lam RW, Milev R, Rotzinger S, Frey BN, Soares CN (2021). Machine learning in the prediction of depression treatment outcomes: a systematic review and meta-analysis. *Psychological Medicine*.

[14] Xiong S, Tian C, Shao M, Liu C (2025). Persistent Airflow Limitation Prediction and Risk Factor Analysis Among Asthmatic Children: A Retrospective Cohort Study. *Pediatric Pulmonology*.

[15] Zhang K, Han Y, Gu F, Gu Z, Zhao J, Chen J (2023). Association between dietary zinc intake and Helicobacter pylori seropositivity in US adults: National Health and Nutrition Examination Survey. *Frontiers in Nutrition*.

[16] Xie Y, Zhang J, Jin X, Liu S, Song W (2024). Development and validation of a nomogram for predicting heterotopic ossification following spinal cord injury. *Clinical Neurology and Neurosurgery*.

[17] Kroenke K, Spitzer RL, Williams JB (2001). The PHQ-9: validity of a brief depression severity measure. *Journal of General Internal Medicine*.

[18] American Psychiatric Association (1994). *Diagnostic and Statistical Manual of Mental Disorders, 4th ed. (DSM-IV)*.

[19] Jorgensen D, White GE, Sekikawa A, Gianaros P (2018). Higher dietary inflammation is associated with increased odds of depression independent of Framingham Risk Score in the National Health and Nutrition Examination Survey. *Nutrition Research (New York, N.Y.)*.

[20] Lai Y, Zhang X, Dong H, Li M (2024). The interaction effects between depression and sleep status on asthma: a national cross-sectional study. *Frontiers in Psychiatry*.

[21] Guo Y, Yan J (2024). Association between asthma and depression: results from the NHANES 2005-2018 and Mendelian randomization analysis. *Postgraduate Medical Journal*.

[22] Chen J, Cheng Z, Yao Y, Wang S (2024). Variation of All-Cause Mortality with Fat-Free Mass Index (FFMI) and Fat Mass Index (FMI) in Individuals with Asthma: Results from the NHANES Database Retrospective Cohort Study. *Journal of Epidemiology and Global Health*.

[23] Al-Dubai SA, Ganasegeran K, Alshakka M, Renganathan P, Elkalmi R, Manaf RM (2016). Anxiety And Depression Among Asthmatic Patients In Malaysia. *ASEAN Journal of Psychiatry*.

[24] Zhang J, Wei J, Gong Y (2023). Risk factors for comorbid anxiety and depression in patients with bronchial asthma. *International Journal of Psychiatry*.

[25] Alanazi TM, Alghamdi HS, Alberreet MS, Alkewaibeen AM, Alkhalefah AM, Omair A (2021). The prevalence of sleep disturbance among asthmatic patients in a tertiary care center. *Scientific Reports*.

[26] Xiang B, Hu M, Yu H, Zhang Y, Wang Q, Xue F (2023). Highlighting the importance of healthy sleep patterns in the risk of adult asthma under the combined effects of genetic susceptibility: a large-scale prospective cohort study of 455 405 participants. *BMJ Open Respiratory Research*.

[27] BaHammam AS, Kendzerska T, Gupta R, Ramasubramanian C, Neubauer DN, Narasimhan M (2016). Comorbid depression in obstructive sleep apnea: an under-recognized association. *Sleep & Breathing = Schlaf & Atmung*.

[28] Yu W, Gong Y, Lai X, Liu J, Rong H (2023). Sleep Duration and Risk of Depression: Empirical Evidence from Chinese Middle-Aged and Older Adults. *Sustainability*.

[29] Kujubu DA, Aboseif SR (2008). An overview of nocturia and the syndrome of nocturnal polyuria in the elderly. *Nature Clinical Practice. Nephrology*.

[30] Weicker H, Strüder HK (2001). Influence of exercise on serotonergic neuromodulation in the brain. *Amino Acids*.

[31] Bateman ED, Hurd SS, Barnes PJ, Bousquet J, Drazen JM, FitzGerald JM (2008). Global strategy for asthma management and prevention: GINA executive summary. *The European Respiratory Journal*.

[32] Maniscalco M, Vatrella A, Cremona G, Carratù L, Sofia M (2001). Exhaled nitric oxide after inhalation of isotonic and hypotonic solutions in healthy subjects. *Clinical Science (London, England: 1979)*.

[33] Trzcińska H, Przybylski G, Kozłowski B, Derdowski S (2012). Analysis of the relation between level of asthma control and depression and anxiety. *Medical Science Monitor: International Medical Journal of Experimental and Clinical Research*.

[34] Toyama M, Hasegawa T, Sakagami T, Koya T, Hayashi M, Kagamu H (2014). Depression’s influence on the Asthma Control Test, Japanese version. *Allergology International: Official Journal of the Japanese Society of Allergology*.

[35] Strine TW, Mokdad AH, Balluz LS, Berry JT, Gonzalez O (2008). Impact of depression and anxiety on quality of life, health behaviors, and asthma control among adults in the United States with asthma, 2006. *The Journal of Asthma: Official Journal of the Association for the Care of Asthma*.

[36] Tarraf H, Al-Jahdali H, Al Qaseer AH, Gjurovic A, Haouichat H, Khassawneh B (2018). Asthma control in adults in the Middle East and North Africa: Results from the ESMAA study. *Respiratory Medicine*.

[37] Lee KH, Lee HS (2020). Hypertension and diabetes mellitus as risk factors for asthma in Korean adults: the Sixth Korea National Health and Nutrition Examination Survey. *International Health*.

[38] Christiansen SC, Schatz M, Yang SJ, Ngor E, Chen W, Zuraw BL (2015). Hypertension and Asthma: A Comorbid Relationship. *The Journal of Allergy and Clinical Immunology. in Practice*.

[39] Li R, Wang X, Luo L, Yuan Y (2024). Identifying the most crucial factors associated with depression based on interpretable machine learning: a case study from CHARLS. *Frontiers in Psychology*.

[40] Su D, Zhang X, He K, Chen Y (2021). Use of machine learning approach to predict depression in the elderly in China: A longitudinal study. *Journal of Affective Disorders*.

[41] Hatton CM, Paton LW, McMillan D, Cussens J, Gilbody S, Tiffin PA (2019). Predicting persistent depressive symptoms in older adults: A machine learning approach to personalised mental healthcare. *Journal of Affective Disorders*.

[42] Xia F, Li Q, Luo X, Wu J (2022). Machine learning model for depression based on heavy metals among aging people: A study with National Health and Nutrition Examination Survey 2017-2018. *Frontiers in Public Health*.

